# Northern ragweed ecotypes flower earlier and longer in response to elevated CO_2_: what are you sneezing at?

**DOI:** 10.1007/s00442-016-3670-x

**Published:** 2016-06-18

**Authors:** Kristina A. Stinson, Jennifer M. Albertine, Laura M. S. Hancock, Tristram G. Seidler, Christine A. Rogers

**Affiliations:** 1 Harvard Forest, Harvard University, 324 North Main Street, Petersham, MA 01366 USA; 2 Department of Environmental Conservation, University of Massachusetts, Amherst, MA 01003 USA; 3 Biology Department, University of Massachusetts, 611 North Pleasant Street, Amherst, MA 01003 USA; 4 Environmental Health Sciences, University of Massachusetts, Amherst, MA 01003 USA

**Keywords:** Ecotypes, CO_2_, Phenology, Plant, Ragweed

## Abstract

Significant changes in plant phenology and flower production are predicted over the next century, but we know relatively little about geographic patterns of this response in many species, even those that potentially impact human wellbeing. We tested for variation in flowering responses of the allergenic plant, *Ambrosia artemisiifolia* (common ragweed). We grew plants originating from three latitudes in the Northeastern USA at experimental levels of CO_2_ (400, 600, and 800 µL L^−1^). We hypothesized that northern ecotypes adapted to shorter growing seasons would flower earlier than their southern counterparts, and thus disproportionately allocate carbon gains from CO_2_ to reproduction. As predicted, latitude of origin and carbon dioxide level significantly influenced the timing and magnitude of flowering. Reproductive onset occurred earlier with increasing latitude, with concurrent increases in the number of flowers produced. Elevated carbon dioxide resulted in earlier reproductive onset in all ecotypes, which was significantly more pronounced in the northern populations. We interpret our findings as evidence for ecotypic variation in ragweed flowering time, as well in responses to CO_2_. Thus, the ecological and human health implications of common ragweed’s response to global change are likely to depend on latitude. We conclude that increased flower production, duration, and possibly pollen output, can be expected in Northeastern United States with rising levels of CO_2_. The effects are likely, however, to be most significant in northern parts of the region.

## Introduction

Changes in flowering time are among the most apparent and widely reported ecological responses of plants to climate change. Many species are flowering earlier with increasingly warmer temperatures (Anderson et al. 2012; Confalonieri et al. 2007; Fitter and Fitter 2002; Huynen and World Health Organization 2003; Rogers et al. 2006; Springer and Ward 2007; Ziska et al. 2011). The greenhouse gas, carbon dioxide (CO_2_) not only alters climate, but also stimulates photosynthesis (Ainsworth et al. 2002; Bazzaz 1990). Though the effects of elevated CO_2_ on plant physiology can often extend to changes in phenology (Leakey et al. 2009; Long et al. 2004), fewer studies have focused on CO_2_ and flowering time and subsequent total flower production (Reekie et al. 1994; Springer and Ward 2007).

Flowering time affects total plant reproductive output, and has direct and indirect consequences for ecological processes such as species interactions, mating success, gene flow, and dispersal (Bolmgren and Cowan 2008; Fitter and Fitter 2002; Primack and Kang 1989; Stinson et al. 2011). Thus, shifts in plant phenology can ultimately affect future plant performance, fitness, and species distributions. However, predicting shifts in the timing and magnitude of flower production requires knowledge of existing genetic and phenotypic variation across a species’ geographic range, and not all populations are expected to respond in the same way (Valladares et al. 2014; Wulff and Alexander 1985). For example, earlier onset of flowering within the life cycle is a typical adaptation to short growing seasons and other harsh conditions among northern populations, while southern populations of the same species may postpone flowering until later in the life cycle to maximize size and reproductive success (Bolmgren and Cowan 2008; Dickerson and Sweet 1971; Grime 1977; Reekie and Bazzaz 1987).

Differentiation into ecotypes with contrasting flowering times is commonly attributed to tradeoffs between growth and reproduction in long and short growing seasons (Dickerson and Sweet 1971; Li et al. 2015; Reekie et al. 1994; Wulff and Alexander 1985). Since elevated CO_2_ offsets resource limitation by enhancing a plant’s carbon pools (Ainsworth et al. 2002), we hypothesized that northern ecotypes adapted to shorter growing seasons would flower earlier than their southern counterparts, and thus disproportionately allocate carbon gains from CO_2_ to reproduction.

Specifically, we hypothesized that northern ecotypes of the widespread ruderal plant, *Ambrosia artemisiifolia* (common ragweed) would disproportionately increase flower production when exposed to higher CO_2_ levels, due to an earlier onset of reproduction compared to southern ecotypes. Common ragweed is found in a vast array of disturbed habitats and produces highly allergenic pollen; 45 % of allergy patients test positive for ragweed allergy (Rogers 2001) and ragweed pollen accounts for ~90 % of fall airborne pollen allergens (Durham 1931; Ziska et al. 2003). In experimental settings, elevated CO_2_ increases relative reproductive effort in ragweed (Rogers et al. 2006; Stinson and Bazzaz 2006) and can increase pollen production by as much as 60 % (Wayne et al. 2002; Ziska and Caulfield 2000).

Ragweed ecotypes have been identified at a broad geographic scale, with earlier flower initiation, shorter flowering window, and smaller plants at high latitudes and later flower initiation and larger plants at lower latitudes across North America (Dickerson and Sweet 1971). A latitudinal phenomenon has also been observed in ragweed pollen counts as a function of temperature changes over the past decade, with areas above the 44°N latitude exhibiting 13–27 days increase in pollen season duration since 1995, using data from the National Allergy Bureau (Ziska et al. 2011). However, the geographic areas covered by these datasets are very broad. Moreover, the effects of CO_2_ on ragweed phenology have not been examined at the regional scale, nor have there been population-level studies on the range of effects of CO_2_ on the flowering response. Such data are necessary to develop accurate trait-based ecological models of future plant species distributions (Anderson et al. 2012; Confalonieri et al. 2007; Valladares et al. 2014), and to interpret landscape ecological patterns at a spatial scale that is relevant to society. From an ecological standpoint, characterizing differences among populations is best done at a regional scale, where the range of environmental heterogeneity is sufficient to detect population-level dynamics and yet within reasonable spatial bounds for meaningful biological interpretation. Understanding regional phenological shifts in this species can thus improve predictions of the full effects of climate change on plant distribution processes, as well as on human health (Confalonieri et al. 2007; Huynen and World Health Organization 2003).

To improve regional predictions of future ragweed pollen production in the Northeastern USA, we focused on three phenotypic aspects that would be important indicators for pollen production: (1) phenology, (onset and duration of male flowers); (2) number of male reproductive structures; and (3) plant architecture (height and branching), which can affect pollen dispersal in wind pollinated plants.

## Materials and methods

### Seed collection and population information

We focused on the greater New England region of the northeastern United States, where temperature varies by as much as 4 °C across latitudinal (north/south) and altitudinal (lowland/upland) gradients (Daly et al. 2002; Ollinger et al. 1995). Urbanization generally declines at higher latitudes, with New York City near the southern edge, being the most urban and populated, and Burlington, Vermont at the northern edge, being lesser populated and surrounded by a rural landscape. Precipitation is about 100–152 cm annually across the region and is less varied across these gradients (Daly et al. 2002).

Seeds were collected from 15 to 20 mature plants in each of 24 wild populations along a climate gradient created with PRISM climate data. We used GIS land cover information to identify ragweed habitats, and randomly selected eight collection points in areas surrounding each of the following three cities: New York, New York (40.71°N, −74.00°W), Boston, Massachusetts (42.35°N, −71.06°W), and Burlington, Vermont (44.48°N, −73.21°W), which we refer to here as low-latitude, mid-latitude, and high-latitude, respectively. To maximize the human-health relevance of our research we chose to study populations near urban centers from distinct points along the climate gradient.

### Fumigation chambers

Twelve enclosed temporary plant growth chambers (each 3.5 m wide × 6 m long) were built of lumber, PVC tubing, and clear plastic sheeting in a 30 × 30 m cleared plot at the Harvard Forest (Petersham, MA, USA). The chambers were spaced about 10 m apart in all directions in a staggered array. Carbon dioxide was supplied from tanks located on site. Four chambers each were randomly assigned to 400 µL L^−1^ (ambient), 600, and 800 µL L^−1^ treatments. Carbon dioxide levels were monitored in the chambers by a computer interfaced CO_2_ sampling and injection array. A Li-Cor 840 infrared gas analyzer sampled air from each chamber at 5-min intervals to determine CO_2_ levels. Injection of CO_2_ into the chamber occurred as necessary via solenoid valve controllers linked to the computer system and CO_2_ supply tanks to maintain levels to ±50 µL of the desired set points. Pots were watered to saturation every five days by automatic drip irrigation system. Automatic temperature controlled vents regulated temperature to within 1.5 °C of ambient temperatures which ranged from 22.4 to 27.9 °C day/12–16.1 °C night. Monthly averages for PAR ranged from 26.1 to 46.2 mol/m^2^.

### Plant propagation

Seeds from each collection site were placed in quartz sand and subjected to a 14-h day length and a temperature of 6 °C day and 4 °C night for 16 weeks, according to stratification methods presented by Pickett and Baskin (1973).

Each of the 24 populations was replicated 4 times within each chamber, resulting in 96 pots per chamber. Five stratified seeds from each location were planted into 4 L pots in the spring of 2011. Each pot was filled with Sunshine Mix #1 (Sun Gro Horticulture, Inc, Bellevue, WA, USA). Ten days after germination commenced, the pots were thinned to one plant per pot.

Throughout the growing season, pots were watered to saturation every 2–4 days as needed to maintain ample and even moisture in all of the treatments. We fertilized the plants using slow-release nitrogen:phosphorous:potassium 14:14:14 (Osmocote; Scott’s, Marysville, OH, USA), reflecting nutrient conditions within an ideal growth range for this species.

### Plant measurements

Plants were assessed weekly for germination, height, branching, and number of buds and flowers during the first 9 weeks of the life cycle. After 9 weeks plants were monitored for presence or absence of flowers, and observations continued until senescence. The following phase lengths were calculated from the data: pre-flowering phase (number of days from germination to flowering); life cycle length (number of days from germination to senescence); and flowering phase (number of days from first bud appearance to observation of last living flower, which coincided with whole plant senescence).

### Statistical analysis

We constructed a two-way analysis of variance model with interactions to test for treatment and latitudinal effects on phase lengths. CO_2_ treatment and latitude of origin were specified as main effects and the CO_2_ × latitude interaction term was included as a test or ecotypic variation in the CO_2_ response. Chamber was included as a nested factor within CO_2_ treatment. We used a separate analysis of covariance model to test for treatment and latitude effects on plant morphological development and flower production through time. In this test, CO_2_ treatment and latitude of origin were specified as explanatory main effects, with chamber nested within treatment, and time (date of weekly observation) specified as a covariate. An individual plant term was included as a random effect in the model. The morphological variables were transformed prior to analysis. Height data were log transformed; number of male reproductive structures and number of branches were square root transformed prior to the analysis. All analyses were completed using JMP 11 (SAS Institute Inc, North Carolina, USA).

## Results

### Phenology

**Fig. 1:**
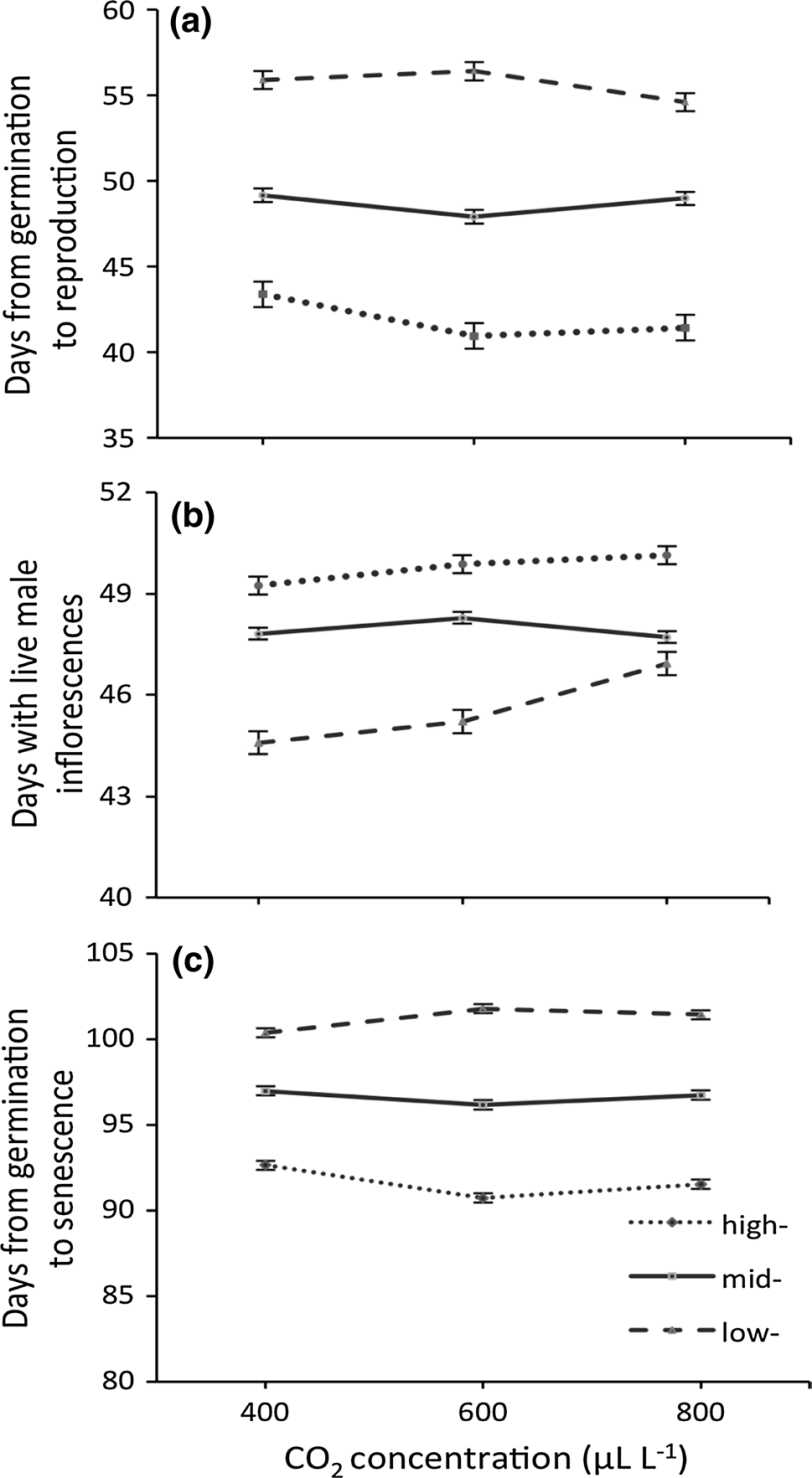
Effects of CO_2_ concentrations and latitude on **a** time to start of flowering; **b** time of active production of male flowers; **c** duration of life cycle. Time is measured in number of days and values are mean ± SE

Both latitude of origin and CO_2_ treatment had significant effects on the timing of flowering. Onset of flowering occurred ~10 days earlier in plants from higher latitude compared to low latitude across all treatments (Fig. 1a, effect of latitude on pre-flowering phase, Table 1). Elevated carbon dioxide resulted in ~2–3 day earlier onset of flowering on average (effect of CO_2_ on pre-flowering phase, Table 1), but plants from different latitudes varied in their response to the treatments (latitude × CO_2_ interaction, Table 1). Plants from the high latitudes flowered ~2.5 days more rapidly in response to intermediate levels of increased CO_2_ in the high-latitude populations, and plants from the low latitude flowered ~1.5 days more rapidly in the high CO_2_ treatment (Table 1, latitude × CO_2_ effect on time to reproduction). There was a significant effect of latitude on length of the flowering phase, with high-latitude populations flowering ~5 days longer on average than those from low latitudes and 2–3 days longer than those from mid latitude (Fig. 1b; Table 1). In contrast, the length of the life cycle was consistently ~7 days longer amongst plants from low latitudes than those from high latitudes and ~4 days longer than those from mid latitudes (Fig. 1c; Table 1), such that high-latitude-sourced plants demonstrated a shorter life cycle and flowered for a longer proportion of the life cycle than plants sourced from mid- or low-latitudes. As indicated by significant CO_2_ × Latitude interaction, the effect of CO_2_ on length of life cycle was found to vary by ~1–2 days across latitudes, increasing in low latitude plants and decreasing in high latitude plants.

**Table 1:** Effects of CO_2_ treatment, Latitude of origin, and CO_2_ × latitude interactions on phase lengths (number of days from: germination to flowering; onset of flowering to senescence; germination to senescence)

Source	*df*	*df* den	*F*	Prob > *F*
Length of pre-flowering phase				
CO_2_	2	1108	4.358	0.013
Latitude	2	1108	508.41	<0.001
Chamber(CO_2_)	9	1108	3.079	0.001
Latitude × chamber(CO_2_)	18	1108	2.02	0.007
CO_2_ × latitude	4	1108	3.11	0.015
Length of flowering phase				
CO_2_	2	1106	2.513	0.081
Latitude	2	1106	37.9	<0.001
Chamber(CO_2_)	9	1106	3.513	0.001
Latitude × chamber(CO_2_)	18	1106	2.45	0.001
CO_2_ × latitude	4	1106	1.312	0.263
Length of life cycle				
CO_2_	2	1100	0.7894	0.454
Latitude	2	1100	327.31	<0.001
Chamber(CO_2_)	9	1100	2.71	0.004
Latitude × chamber(CO_2_)	18	1100	2.33	0.001
CO_2_ × latitude	4	1100	3.572	0.007

For morphological traits, see Table 2

### Growth and morphology

**Fig. 2:**
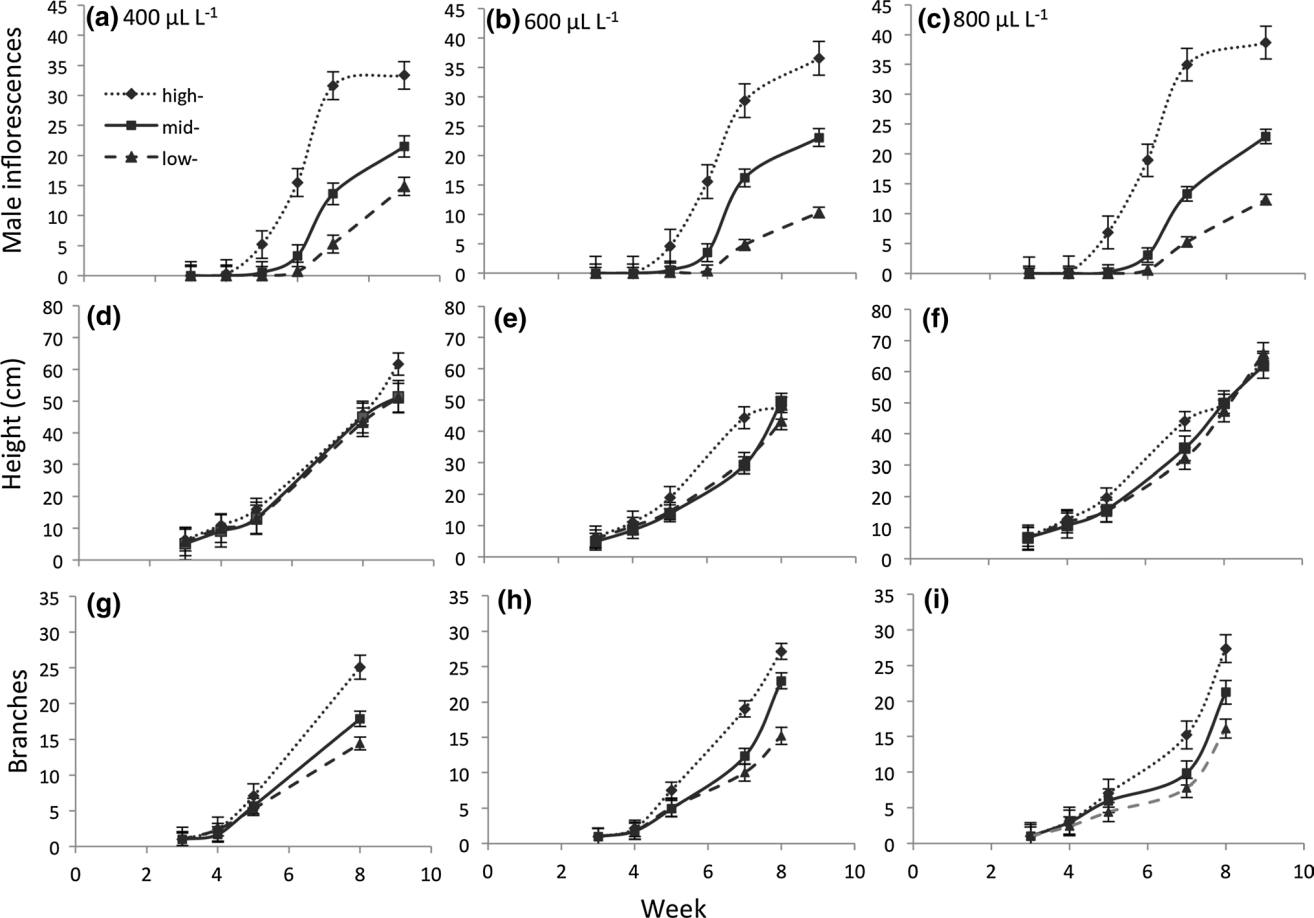
Effects of CO_2_ concentrations and latitude on plant morphological traits over a 10 week study period. Morphological traits include **a**–**c** number of male reproductive structures, **d**–**f** height, and **g**–**i** number of branches. Values are mean ± SE

**Fig. 3:**
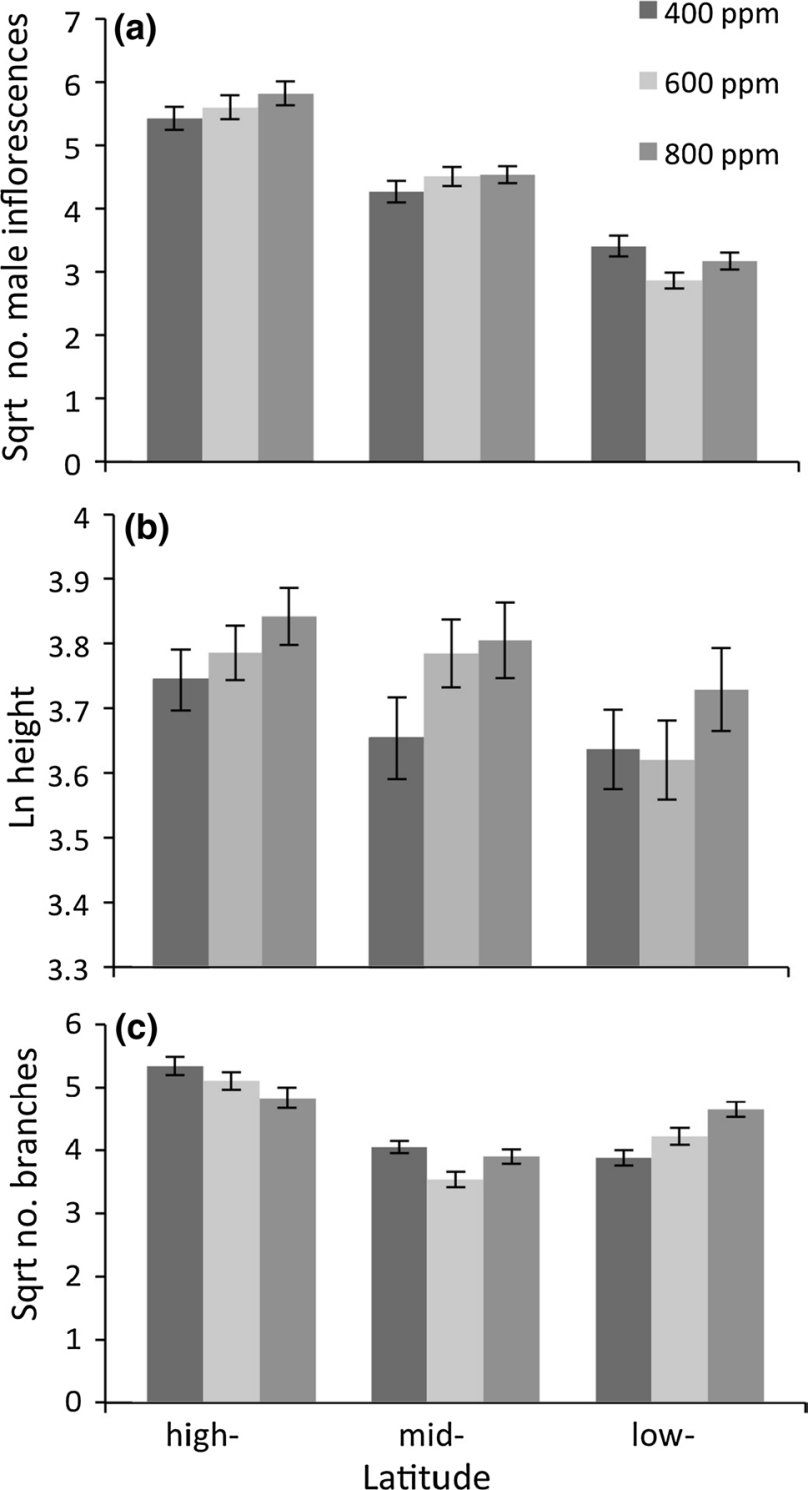
Effects of CO_2_ concentrations and latitude on final plant morphological traits: **a** number of male inflorescences, **b** height in cm, and **c** number of branches. Note that all of the traits were transformed (see “Materials and Methods” for detail) and values shown here are mean ± SE

The production of male reproductive structures was dependent on latitude of origin, and plants from different latitudes varied in the response to CO_2_ treatments (effects of latitude and CO_2_ × latitude, Table 2); high-latitude populations produced the most structures at the fastest rate, followed by mid- and then low- latitudes (Fig. 2a–c; effect of time × latitude and time × co_2_ × latitude, Table 2). Plants from higher latitudes also produced the greatest final number of male reproductive structures in all treatments (Fig. 3a).

**Table 2:** Effects of CO_2_ treatment, latitude of origin, and CO_2_ × latitude interactions on plant morphological traits measured over time (week of observation)

	*df*	*df* den	*F*	Prob > *F*
Number of male inflorescences				
CO_2_	2	1120	2.09	0.124
Latitude	2	1120	583.01	<0.001
CO_2_ × latitude	4	1120	2.56	0.037
Time	5	5526	3642.91	<0.001
Time × CO_2_	10	5525	1.33	0.207
Time × latitude	10	5526	241.62	<0.001
Time × CO_2_ × latitude	20	5525	2.36	0.001
Chamber(CO_2_)	9	1119	13.22	<0.001
Chamber (CO_2_) × latitude	18	1119	3.99	<0.001
Chamber(CO_2_) × time	45	5522	7.22	<0.001
Chamber(CO_2_) × latitude × time	90	5522	2.68	<0.001
Height (cm)				
CO_2_	2	1282	11.63	<0.001
Latitude	2	1301	14.63	<0.001
CO_2_ × latitude	4	1284	0.20	0.939
Time	1	3066	9836.34	<0.001
Time × CO_2_	2	3073	9.36	<0.001
Time × latitude	2	3066	7.54	0.001
Time × CO_2_ × latitude	4	3073	2.63	0.033
Chamber(CO_2_)	9	1220	4.66	<0.001
Chamber (CO_2_) × latitude	18	1222	0.71	0.806
Chamber(CO_2_) × time	9	3072	6.19	<0.001
Chamber(CO_2_) × latitude × time	18	3072	1.60	0.053
Number of branches				
CO_2_	2	1776	6.11	0.002
Latitude	2	1625	56.41	<0.001
CO_2_ × latitude	4	1549	6.06	<0.001
Time	1	2904	6851.52	<0.001
Time × CO_2_	2	2927	6.03	0.002
Time × latitude	2	2886	41.38	<0.001
Time × CO_2_ × latitude	4	2913	12.13	<0.001
Chamber(CO_2_)	9	1459	2.04	0.032
Chamber (CO_2_) × latitude	18	1342	3.48	<0.001
Chamber(CO_2_) × time	9	2925	5.80	<0.001
Chamber(CO_2_) × latitude × time	18	2909	6.48	<0.001

Carbon dioxide level and latitude of origin affected height (effects of CO_2_, latitude, Table 2). The latitude effect on height varied with time, and the effect of CO_2_ on plant height depended on latitude of origin (latitude × time, and latitude × time × CO_2_, Table 2). High-latitude populations achieved greater height earlier in the growing season than mid- and low-latitude populations, and this effect was magnified at elevated CO_2_ (Fig. 2d–f). At the final height measurement, plants from higher latitudes had taller plants and a trend toward increased height with higher CO_2_ levels in all source latitudes (Fig. 3b).

Latitude of origin had significant effects on branching in ragweed plants (latitude, Table 2), and CO_2_ impacts on branch number was dependent on latitude (CO_2_ × latitude: Table 2). Branching was increased by elevated carbon dioxide in the low-latitude populations and decreased in the high-latitude populations, while it had no effect on branching in mid-latitude populations (Fig. 3c). However, low-latitude populations had the most branching regardless of CO_2_ level (Fig. 2c). Both latitude and CO_2_ effects were dependent on time (latitude × time; CO_2_ × time; Table 2) with both having a significant impact on branching later in the life cycle (Fig. 2g–i).

### Chamber effects

The nested factor, chamber, contributed significantly to the variation observed in most of the measured variables. However, we found no evidence to suggest that any specific chamber or chambers accounted for the main effects of CO_2_ or latitude or their interactions in the model or for any given plant response; mean responses were similar across all chambers.

## Discussion

As predicted, we identified latitudinal ecotypes in common ragweed in the Northeastern USA with divergent patterns of growth, flowering time and flower production, as well as responses to elevated CO_2_. Specifically, plants from northern latitudes produced more flowers as a result of longer and earlier flowering periods, and these traits tended to be exaggerated at elevated levels of CO_2_. Plants from higher latitudes were also taller and produced more branches, both of which have been shown to correlate with increased reproductive effort and pollen dispersal distances (Bolmgren and Cowan 2008; Dickerson and Sweet 1971; Lloyd and Bawa 1984). In contrast, plants from the southern part of the region produced relatively fewer branches and flowers, and spent more of their life cycle in vegetative growth. Moreover, elevated CO_2_ caused earlier flowering in plants from northern latitudes, which was also accompanied by markedly increased total male flower production.

Although southern plants responded to elevated CO_2_ by branching more and flowering earlier, they did not increase their flower production. Instead, photosynthetic gains from higher CO_2_ levels were partitioned primarily to growth, as has been observed in some ragweed genotypes from a single population (Stinson et al. 2011). Because plants from different latitudes maintained phenotypic differences in phenology and morphology when grown at a common latitude and across CO_2_ treatments, we conclude that there is some genetic control over these traits reflecting different ragweed ecotypes (Clausen et al. 1948). Thus, it is likely that responses of ragweed to rising levels of CO_2_ will differ across the Northeastern USA and will not be consistent across the region, but rather will be disproportionately stimulated in northern latitudes.

The ecotypic divergence we observed appears to reflect local adaptations that are mediated by phenology. As has been shown in classical ecological studies, rapid reproduction is often favored at higher latitudes where short growing seasons and the risk of early frost create a strong selective pressure to reach reproduction before the end of the life cycle (Clausen et al. 1948). Under milder conditions at lower latitudes, delayed reproduction may be important to maximize size at the time of flowering, protect against natural enemies, and compete for resources (Bazzaz et al. 1987; Bazzaz et al. 1995; Grime 1977; Reekie and Bazzaz 1987; Stinson et al. 2006). As we predicted, our data indicate that CO_2_ disproportionately increases flowering in northern ecotypes because their life history favors rapid reproduction; carbon gains are allocated preferentially to earlier onset and increased production of flowers. Although we did not test for underlying physiological controls over flowering, one possibility is that ecotypes differ in carbon:nitrogen balance, which can alter cellular sugar signaling to initiate flowering (Coruzzi and Zhou 2001; Rolland et al. 2006).

Because the CO_2_ effects on flowering were most pronounced in plants from the mid- and high- latitudes, our findings suggest that future environmental conditions may have a magnified effect on pollen production in the more northern areas, resulting in the potential for increased reproductive output at higher latitudes of the study region. However, the slight lengthening of the flowering phase in lower latitude populations may result in extended production of pollen, thereby partially offsetting the CO_2_-enhancements to total flower production in plants from higher latitudes. Our work highlights the importance of population-level variation as posed by (Valladares et al. 2014) in predicting species responses to ongoing global changes, and also provides an ecological context for the broader latitudinal patterns of enhanced reproduction and longer flowering duration in northern parts of the species’ geographic range (Ziska et al. 2011). Our observations of ecotypic variation in CO_2_ response across the Northeastern United States represents a relatively small geographic area. Others report ecotypic variation in flowering and size at broader spatial scales in this species, suggesting that variation in the CO_2_ response would hold and perhaps be amplified at broader spatial scales.

Because temperatures are increasing more quickly and dramatically at higher latitudes (Confalonieri et al. 2007; Ziska et al. 2011) it is likely that the combined effects of CO_2_ and warming will continue to act synergistically to increase pollen production, height, and branching of ragweed plants in northern areas. Disproportionate allocation to reproduction could lead to relatively greater reproductive success and pollen production by northern ecotypes of common ragweed. We, therefore, extend the ecological outlook for this species to include higher reproductive and outcrossing rates, and perhaps a skewed increase in gene flow, at higher latitudes. The ecological implications could include increased dominance of early flowering genotypes at the landscape scale (Valladares et al. 2014). Moreover, paleo-ecological records suggest that landscape disturbances and warm/dry conditions are correlated with significant historical increases in ragweed pollen and abundance across Northeast United States (Faison et al. 2006). Future moderation of environmental conditions due to warming and longer growing seasons in higher latitudes, in combination with ever-increasing development and landscape disturbances are therefore likely to favor increasing ragweed presence.

In conclusion, our data provide much needed resolution and mechanistic understanding of variation in plant responses to global change. We not only show that elevated CO_2_ will extend ragweed phenology and flower production in the Northeastern United States, but we capture ecotypic differences among populations that correspond with environmental heterogeneity within this region. We emphasize that total flower production may be enhanced in the northernmost areas by increasing levels of CO_2_, due largely to underlying proclivity towards rapid reproduction and longer flowering periods in northern ecotypes. Since ragweed pollen has a negative impact on public health, our data should raise concerns and awareness about disproportionate effects on different human populations, even within a relatively small geographic region.
